# Natural dyes developed by microbial-nanosilver to produce antimicrobial and anticancer textiles

**DOI:** 10.1186/s12934-024-02457-3

**Published:** 2024-07-02

**Authors:** Osama M. Darwesh, Ahmed Marzoog, Ibrahim A. Matter, Mohammad K. Okla, Mohamed A. El-Tayeb, Mohammed Aufy, Turki M. Dawoud, Mostafa A. Abdel-Maksoud

**Affiliations:** 1 Agricultural Microbiology Department, National Research Centre, Dokki, Cairo, 12622 Egypt; 2 Department of Soil and Water Sciences, College of Agriculture, University of Anbar, Ramadi, Iraq; 3 Botany and Microbiology Department, College of Science, King Saud University, Riyadh, Saudi Arabia; 4 Department of Pharmaceutical Sciences, Division of Pharmacology and Toxicology, University of Vienna, Vienna, Austria

**Keywords:** Nanocomposite, Antimicrobial natural pigment, *Streptomyces torulosus*, Special textiles, Skin cancer

## Abstract

Developing special textiles (for patients in hospitals for example) properties, special antimicrobial and anticancer, was the main objective of the current work. The developed textiles were produced after dyeing by the novel formula of natural (non-environmental toxic) pigments (melanin amended by microbial-AgNPs). *Streptomyces torulosus* isolate OSh10 with accession number KX753680.1 was selected as a superior producer for brown natural pigment. By optimization processes, some different pigment colors were observed after growing the tested strain on the 3 media. Dextrose and malt extract enhanced the bacteria to produce a reddish-black color. However, glycerol as the main carbon source and NaNO_3_ and asparagine as a nitrogen source were noted as the best for the production of brown pigment. In another case, starch as a polysaccharide was the best carbon for the production of deep green pigment. Peptone and NaNO_3_ are the best nitrogen sources for the production of deep green pigment. Microbial-AgNPs were produced by *Fusarium oxysporum* with a size of 7–21 nm, and the shape was spherical. These nanoparticles were used to produce pigments-nanocomposite to improve their promising properties. The antimicrobial of nanoparticles and textiles dyeing by nanocomposites was recorded against multidrug-resistant pathogens. The new nanocomposite improved pigments' dyeing action and textile properties. The produced textiles had anticancer activity against skin cancer cells with non-cytotoxicity detectable action against normal skin cells. The obtained results indicate to application of these textiles in hospital patients’ clothes.

**Supplementary Information:**

The online version contains supplementary material available at 10.1186/s12934-024-02457-3.

## Introduction

Synthetic dyes are produced primarily from non-renewable petrochemicals, have complex chemical structures, and have many uses in various fields [1]. More than 800,000 tons/year are produced globally as industrial dyes, about three-quarters of which are used in the textile industry [2]. Growing environmental concerns have prompted researchers and scientists to study the hazardous effects of synthetic dyes on aquatic and terrestrial environments. Throughout the dyeing procedures, the usual synthetic dye isn’t fixed onto the textiles and is released to industrial wastewater ending in the environment about 70–90% [3]. Also regarding environmental impacts, researchers have noted potential negative impacts on the public health of workers in this industry and/or users of its end products [4]. Exposure to synthetic dyes can hurt human health, especially causing respiratory or skin allergies [5]. Therefore, researchers work hard to find alternative colors for synthetic dyes. Natural dyes are eco-friendly, renewable resources, and biodegradable materials produced from organisms and can give colors to many materials. They are generally produced by plants, animals, and microbes [6, 7]. The demand for high amounts of natural dyes has led to finding new sources for dye producers [8]. Among them, microbial pigments have been taken the attention of numerous authors based on their rapid growth in cultural media, diversity, larger productivity, being limited by time or place, ease of controlling the fermentation process and feasible for cultivation using cost-effective substrates, especially agro-industrial wastes [9]. Microbial pigments can be produced by bacteria, action-bacteria, fungi, yeasts as non-filament fungi, and microalgae either using submerged fermentation or solid substrate fermentation technology because advancements in fermentation technologies result in easy production and separation of dyes [10]. Most public bacterial dyes are melanins, carotenoids, quinones, phenazines, tambjamines, violacein, and prodigiosin [11]. These pigments are effective as UV protectants and antioxidants, so they have many potential health and medical applications as anticancer, antimalarial, and antimicrobial substances [12]. The most common pigments from action bacteria were melanins (colors, black, brown, olive), carotenoids (red, yellow, pink, violet), and blue pigments related to actinorhodins [13]. Numerous novel nanoparticles have been measured for a diverse class of fabrics and they have shown great potential in developing color and several functional goods namely self-cleaning, antimicrobial, ultraviolet (UV) protection, hydrophilic, and so on [14]. Depending on silver nanoparticle properties, the cellulosic fabric was finished via silver nanoparticles to grow antimicrobial properties and to increase its artistic worth [15]. The silver-modified cotton fabric showed over 99% bacterial reduction especially *Staphylococcus aureus* and *Escherichia coli* with a content on cotton by 88 mg/kg. The antimicrobial action of the silver-treated cotton fabric was preserved at over 98% reduction after being exposed to 20 consecutive home laundering conditions [16].

Bacterial poisons are a constant danger to the human community due to their fast spread and capability to mutate rapidly, in addition to be resistance toward antibiotics. Hereafter, researchers and medical scientists have developed novel antimicrobials, like bacterial pigments to fight antibiotic resistance. In this case, the red pigment of *Streptomyces* sp. is exposed to deliver acceptable protection action against *Bacillus* and *Micrococcus* [17]. The genus of *Streptomyces* can produce antibacterial pigments like actinorhodin and tetracycline [18]. Tetracycline is an advantage by already an FDA-approved drug, and the antibacterial molecule delays the pathogen’s protein creation. Also, a green-colored pigment is produced by *Bacillus cereus* (cerein) and it has a bactericidal action against other *Bacillus cereus* strains [19]. Recent soundings have been led to study the bactericidal potential of bacterial pigments from halophilic environments. The natural pigmenting products have no side effects on the customer, making them more needed for large-scale bactericidal manufacturing. Pyocyanin also holds anti-bacterial actions in contradiction of numerous Gram-negative and Gram-positive bacteria like *E. faecalis*, *St. aureus, St. saprophyticus, K. pneumonia*, *Morganella morganii* and *Proteus mirabilis* [11, 20]. Thus, the present work objectived to develop natural dyes nanocomposites as a novel technology for dyeing special clothes for human healthcare in hospitals. Also, production of effective natural dyed fabrics to inhibit pathogens' growth and skin cancer cell development was the main goal of the current research.

## Materials and methods

### Actinobacterial strain for natural pigments production

*Streptomyces torulosus* strain OSh10 with accession number, KX7536801 was isolated from a soil sample at National Research Centre garden, Cairo, Egypt. All details about this strain were presented in previous work [21]. The actino-bacterial strain was used as a source of natural dyes production using three media (yeast malt, tyrosine, and glycerol/asparagine), the composition of these media was previously shown by Darwesh et al. [11].

### Optimization of natural dye production using different carbon and nitrogen sources

Five carbon sources were used in this study to enhance pigments production. The type of sugar was selected depending on the kind of production media (Table S1). The carbon source was selected as mono, di and poly saccharide formulas. For optimization of nitrogen source, five different nitrogen sources were applied. The main and substituted nitrogen source was tabulated in Table (S1). Also, the nitrogen source was selected as organic nitrogen, mineral nitrate, and mineral ammonia. A volume of 100 ml of media with different carbon sources was placed in 250-mL Erlenmeyer flasks and inoculated with a 1-cm-diameter disk of mycelia and spores of *Streptomyces* sp. The action-bacterial disc was obtained from a five-day-old culture grown on starch nitrate agar plates. The action-bacteria-inoculated Erlenmeyer flasks were incubated for seven days in an orbital incubator shaker at 120 rpm, 30 °C. Samples were collected daily and the produced dye(s) were measured by spectrophotometer at the appropriate wavelength.

### Production of different pigments using different broth media for dyeing processes

Eight different dye production media were obtained in the previous section because they had some other pigments. One desk (1 cm diameter) of *Streptomyces torulosus* was inoculated into 500 mL flasks containing 200 mL of each optimized modified medium and then incubated in an incubator shaker (120 rpm) at 30 ºC for 7 days. After dye(s) production, the flasks were filtered and the supernatant was collected for further testing.

### Microbial synthesis and characterization of silver nanoparticles (AgNPs)

The fungus *Fusarium oxysporum* (obtained from the Department of Agric. Microbiology, National Research Centre, Egypt) was used to produce bio-reducing agents for synthesizing nanoparticles from the corresponding metal salts. The fungal strain was activated by streaking onto a potato dextrose agar plate. After 5 days of incubation, one disk (1 cm diameter) was used as an inoculant for potato dextrose broth (250 mL flask containing 100 mL medium). The flask was incubated in an incubator shaker (120 rpm) at 28 ± 2 ºC for 3 days and then filtered using filter paper (Whatman No.1). Using 1 mM silver nitrate, the cell-free fungal supernatant was utilized to synthesize silver nanoparticles. The filtrate was added to silver nitrate solution by a ratio of 1:1 and left at room temperature (25 ºC) for 24 h, in the dark. Following incubation, the produced silver nanoparticles changed color to a dark brown. They were then collected by centrifugation, repeatedly rinsed in deionized water, and dried at 60 ºC.

By using a transmission electron microscope (JEOL; JEM-1400 TEM), the generated nanoparticles were characterized. The samples were prepared by placing a drop of well-dispersed NPs onto 200-mesh amorphous carbon-coated grid and dried at ambient temperature [22].

### Formulation of natural dyes nanocomposites

About 485 µg of AgNPs was added to 100 mL of the produced dyes to formulate nanocomposites. The mixtures were left under shaking conditions for 2.5 h at ambient temperature. After mixing, the formed nanocomposites were characterized using transmission electron microscopy JEOL (JEM-1400 TEM).

### Evaluation of antimicrobial activity

The antimicrobial activity of the produced microbial pigments, biosynthesized AgNPs, or nanocomposite was assayed against common pathogenic microorganisms. The applied pathogens were obtained from the American-type culture collection (ATCC; Rockville, MD, USA). *Staphylococcus aureus* (ATCC-47077), and *Bacillus cereus* (ATCC-12228) as representatives of Gram-positive bacteria, while *Escherichia coli* (ATCC-25922), *Salmonella typhi* (ATCC-15566) were used as representatives of Gram-negative bacteria*.* In addition, *Candida albicans* (ATCC-10231), *Saccharomyces cerevisiae* (ATCC-9763), and *Aspergillus niger* (ATCC-16888) were applied as representatives of yeast and fungal pathogens, respectively in this study.

The agar wells diffusion technique was used to assist the antimicrobial activity of the targeted materials in this study [23, 24]. Briefly, nutrient agar and potato dextrose agar plates were prepared for bacteria and fungi. A 0.1 mL of fresh cultures containing 10^6^ cfu/mL of pathogenic microorganisms (spore suspensions in sporozoite fungi) was used to inoculate agar plates individually. Utilizing a sterile cork-borer in solidified agar, wells of 6 mm in diameter were excavated on the inoculated agar plates. The wells were filled with the tested pigments (about 70 µL), biosynthesized AgNPs (roughly 70 µL of 1000 µg/mL), and nanocomposite (roughly 70 µL). Plates were incubated for 24 h at 37 °C, except *Aspergillus niger*, which was incubated for 72 h at 28 °C after being left for two hours at 4 °C to allow for diffusion. The antimicrobial activities of tested materials were assessed by measuring the three replicates of the inhibitory zones surrounding the well in millimeters [25]. In addition, the antimicrobial activity of dyed textiles either treated by normal dyes or nano-composited dye was examined as an inhibition zone produced around 1 cm desks according to a previous study [26]. The minimum inhibitory concentration (MIC) of AgNPs and/or nanocomposite was also determined using decreasing concentrations on pathogenic agar plates as previously described. MIC was determined using the lowest dosed well's concentration, which did not visually exhibit a zone of inhibition.

### Dyeing of nylon and wood textiles

The nylon 6 single jersey knit fabric (114 g/m^2^, 135 D/30 F) was obtained after pre-cleaning and bleaching from El Shourbagy Company (Egypt). The fabric sample was pre-treated with a prepared solution containing 2 g/L sodium carbonate and 5 g/L nonionic detergent (Hostapal CV, Clariant, Egypt) at 60 °C for 30 min before dyeing. It was then completely cleaned in water and allowed to air dry at room temperature. In addition, Golden Tex Co., Tenth of Ramadan, Egypt, supplied the wool fabric (310 g/m^2^). After being treated for 30 min at 60 °C in an aqueous solution with a liquor ratio of 50:1 that contained 0.5 g/L sodium carbonate and 2 g/L nonionic detergent, it was completely rinsed and allowed to dry at room temperature. Wool and polyamide materials were subjected to lab-scale dyeing trials utilizing the generated dyes without the use of mordants [27]. The generated dye was added to the dye bath at a liquor ratio of 50:1, and the pH was corrected to 3.0 using 1 g/L of amphoteric leveling agent (Albegal B). After 10 min at 50 °C to start the dyeing process, the temperature of the dye bath was increased to a boil and maintained for 45 min. Once the dyeing process was completed, the temperature was lowered to 60 °C. The dyed samples were then rinsed, and cleaned for 30 min at 60 °C with a liquid ratio of 50:1 in an aqueous solution containing 2 g/L nonionic detergent. The dyed and washed fabric samples were air-dried at room temperature and kept for further investigations.

### Characterization of dyed textiles properties

The λ max was first determined for the dye components. Using standard curves of distinct compounds determined by UV/VIS spectrophotometry (Perkin Elmer Double Beam Spectrophotometer, USA), single dye compounds were quantitatively quantified. Regarding color strength, the Kubelka–Munk equation was utilized to measure the reflectance values of the dyed fabrics utilizing a data color SF 600 + Relative color strengths (K/S values) [28].

$$K/S = \frac{{\left( {1 - R} \right)^{2} }}{2R}$$

where K is the absorption coefficient, S is the scattering coefficient, and R is the decimal fraction of the dyed fabric's reflectance.

For the determination of CIE L*, a*, b* value, an organization called International Commission on Illumination abbreviated (CIE), Vienna, Austria as the international authority on light, illumination, color, and color spaces was applied to determine these standard values [28]. The colored samples were assessed for fastness using ISO standard procedures [29]. The three particular tests were: color fastness to light (carbon arc), color fastness to perspiration, and color fastness to washing. The color fastness to washing was determined according to ISO 105-C02 method (1989). The composite specimens were sewed between two pieces of bleached cotton and wool fabrics and then immersed into an aqueous solution containing 5 g/L soap nonionic detergents at a liquor ratio of 50:1. After 45 min of thermostatically controlled bathing at 50 °C, the samples were taken out, rinsed twice with occasional hand squeezing, and dried. The "Gray-scale" was used to evaluate the wash fastness for color change. In the meantime, two artificial perspiration solutions were made in accordance with ISO 105-E04 (1989) for the measurement of color fastness to perspiration. The solutions were basically prepared by dissolving L-Histidine monohydrochloride monohydrate (0.5 g), sodium chloride (5 g), and sodium dihydrogen orthophosphate (2.2 g) in one liter of DW. The pH was adjusted using 0.1 N of sodium hydroxide solution to 5.5 or 8 for acidic and alkaline solutions, respectively. To create a composite specimen, the colored specimen (5 × 4 cm) was sewn between two pieces of the uncolored specimen.

To ensure thorough wetting, the composite sample was submerged in each solution for 15 to 30 min while being occasionally shaken and squeezed. A force of approximately 4–5 kg was applied to position the test specimen between two plates made of plastic or glass. After that, the plates with the composite specimens were kept vertically in an oven set at 37 °C for four hours. The test specimen's color change effect was characterized and expressed in terms of the grey scale. The test for light fastness was conducted using a continuous 35-h xenon light source in compliance with ISO 105-B02, 1988. The blue scale for color change was used to define and express the impact on the test samples' color [30].

### Anticancer activity evaluation and cytotoxicity determination

The human epidermoid carcinoma A431 cell line and human normal fibroblast cell line (BJ1) were applied to evaluate the anticancer activity and cytotoxicity of produced dyed textiles. “Bioassay-Cell Culture Laboratory, National Research Centre (Cairo, Egypt)” was the place for doing the tests. By converting the yellow color of 3-(4,5-dimethylthiazol-2-yl)-2,5-diphenyl tetrazolium bromide (MTT) to purple formazan in a mitochondrial-dependent reaction, cell viability was determined. The neutral red uptake assay was followed for conducting the test [31, 32]. Trypsinized cells were subcultured in tissue culture flasks (25 cm^2^). Each flask held five hundred and five cells, with seven milliliters of full Dulbecco's modified eagle medium (DMEM) supplemented with one percent antibiotic solution (100 U/mL penicillin and 100 μg/mL streptomycin) and ten percent fetal bovine serum then incubated at 37 ± 1 °C. A431 cancer cells as well as normal cells *i.e.* BJ1 were divided into untreated control (Group 1) and inoculated cells with dyed textiles (Group 2). Neutral red dye (4 mg/mL) in serum-free DMEM was added to the cells for three hours. The cells were de-stained using a de-staining solution (5% glacial acetic acid and 50% EtOH in distilled water) after being cleaned with phosphate-buffered saline (PBS). Spectrophotometer measurement for the absorbance at 540 nm of residues neutral red dye was performed. Treatments were performed in triplicate after incubation for 24 h.

### Statistical analysis

The obtained data were statistically analyzed using Origin software, version 8 (Origin Lab). LSD at 5% of means values was compared; the means and standard deviations are illustrated in the figures by Origin software [33].

## Results and discussion

### Optimization of pigment production using different carbon sources

The optimization of the fermentation medium and process conditions is an important step to maximize the benefit of the fermentation process [34]. Microbial secondary metabolites are influenced by environmental and cultural conditions e.g. pH, illumination, carbon, and nitrogen source. In a previous study [21], the selected media for pigment production were yeast-extract malt-extract, glycerol-asparagine, and tyrosine media. The present study aimed to enhance the pigment production using *Streptomyces torulosus* isolated from Egyptian soil and it had a high ability to produce three different pigments such as reddish black, brown and green in yeast malt, tyrosine, and glycerol asparagine media, respectively by changing the carbon and nitrogen sources.

**Fig. 1:**
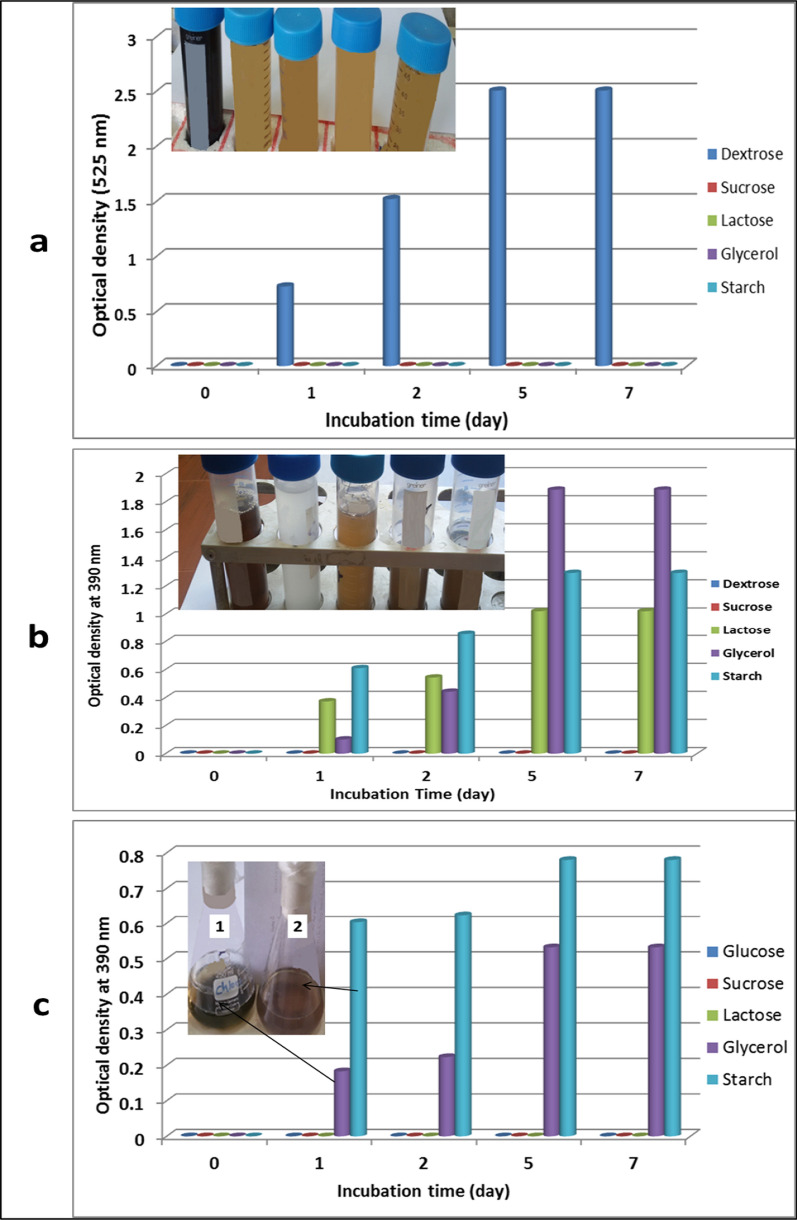
Pigments production after 7 days of incubation using different carbon sources in yeast malt broth (**a**), tyrosine broth medium (**b**) and glycerol asparagine broth (**c**); where **1** is glycerol and **2** is starch in glycerol-asparagine medium

To improve the pigments produced on yeast malt broth medium, more than one carbon source was used. All sugars used in this work belonged to different sugar types (mono, di, and polysaccharide) to study the microbial pathway for pigment production. Dextrose (monosaccharide) as the main carbon source of this medium was substituted by sucrose and lactose as disaccharides and glycerol, and starch as polysaccharides. The results of pigment production were illustrated in Fig. 1a. This result indicated that the dextrose was the best carbon source for production of reddish black pigment. The pigment was produced after one day and developed sequentially until the 7th day of incubation. The development of pigment production reached to 2.506 of absorption.

In the case of tyrosine broth medium (brown pigment), four carbon sources were used to change the main carbon source. After changing glycerol by dextrose, sucrose, lactose, and starch, the samples were collected daily and the produced pigment was measured by spectrophotometer at a wavelength of 390 nm. The obtained data (as shown in Fig. 1b**)** highlighted that glycerol as the main carbon in this medium was the best carbon source for the production of brown pigment. The pigment formation was detected at a one-day-old culture and increased during the seven days of incubation; however, the pigment density was stabilized at a 5-days culture (as its absorbance reached 1.886). In the case of glycerol asparagine broth medium (ISP5), four carbon sources involved in different sugar groups were used to change the main carbon source. Glycerol was substituted by dextrose, sucrose, lactose, and starch, and the samples were collected every day and the production of pigment was measured at a wavelength of 390 nm by spectrophotometer. The results presented in Fig. 1c noted that starch as polysaccharide was the best carbon source for the production of deep green pigment reaching 0.780.

From that, we can conclude that the polysaccharide was considered as initiated pigment production by *Streptomyces torulosus* using glycerol asparagine medium. These obtained results are consistent with those recorded by Hewedy and Ashour [35], who studied pigment production by *Kluyveromyces marxianus* and *Streptomyces chibaensis*. They found that all the different carbon sources used in “glucose yeast extract peptone broth” resulted in good growth and pigmentation production except succinic and malic acid. In addition, when D-xylose was used as a carbon source, the examined microorganisms showed significant growth and pigmentation. Also, Pandey et al. [36], reported that the addition of 2% maltose as a carbon source to potato dextrose broth medium guided *Penicillium* sp. (GBPI-P155) to produce deep natural red pigment.

### Optimization of pigment production using different nitrogen sources

**Fig. 2:**
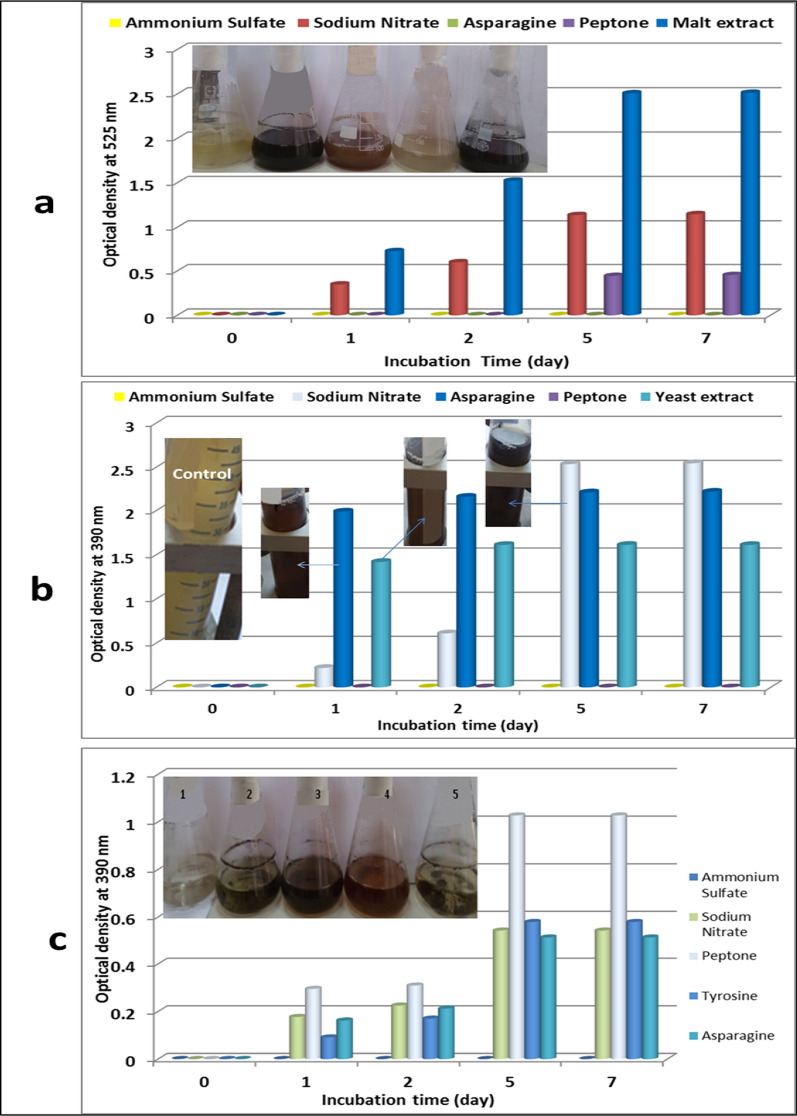
Pigment production using different nitrogen sources in yeast malt broth medium (**a**), tyrosine broth medium (**b**) and glycerol asparagine broth (**c**); where; **1** as ammonium sulphate, **2** as sodium nitrate, **3** as peptone, **4** as tyrosine and **5** as asparagine

Another essential nutrient element is nitrogen, so, it is important to optimize nitrogen sources for pigment production by *Streptomyces torulosus*. The yeast malt broth medium (ISP2) is one of the most important mediums for the production of pigment by *Streptomyces* strains [37]. This medium was used to produce reddish black pigment using *Streptomyces torulosus* strain with malt extract as the main nitrogen source. this nitrogen source was replaced by asparagine, peptone, NaNO_3_, and (NH_4_)_2_SO_4_ (organic and inorganic substrate) to understand the role of nitrogen source in the pigment(s) production process. The results of pigment(s) production were noted every day after measured by spectrophotometer at 525 nm and the samples were collected on the last day were illustrated in Fig. 2a. This result indicated that malt extract and NaNO_3_ were the best nitrogen sources for the production of reddish black and greenish-black pigments, respectively. The pigments reached 2.506 and 1.135 for malt extract and NaNO_3_ as nitrogen source, respectively.

The second medium was the tyrosine medium. To enhance pigment production, four nitrogen sources involved in different nitrogen types were used to change the main nitrogen source of tyrosine broth. Yeast, peptone as organic nitrogen, and NaNO_3_, (NH_4_)_2_SO_4_ as inorganic nitrogen were used instead of asparagine. The results represented in Fig. 2b indicated that NaNO_3,_ asparagine, and yeast extract were responsible for the production of deep brown, brown, and pal brown pigments, respectively. The pigments reached to 2.535, 2.207 and 1.483, respectively. For glycerol asparagine medium, four nitrogen sources, tyrosine, peptone as organic nitrogen, and NaNO_3_, (NH_4_)_2_SO_4_ as inorganic nitrogen were used instead of asparagine. The results represented in Fig. 2c indicated that peptone, tyrosine, and NaNO_3_ were the best nitrogen sources for the production of deep green, brown, and green pigments, respectively. The pigments were produced after one day and developed after that until the 7th day and reached 1.027, 0.579, and 0.540, respectively.

### Production of different natural pigments by *Streptomyces torulosus* and dyeing process

The main objective of the current work was to produce natural pigment(s) with dyeing efficiency. To achieve this aim, *Streptomyces torulosus* was selected from various actino-bacteria as a superior pigment producer and used in the present study under optimization processes. Some different natural pigments were produced based on changing carbon and nitrogen sources of three selected media. The pigments were produced at optimum conditions and used for dyeing two textiles (wool and polyamide 6). The results indicated that all tested pigments had a high ability for dyeing the polyamide and wool fabrics (Table 1). In this work, wool and polyamide fabrics were chosen depending on their beneficial uses in hospitality clothes [38, 39].

**Table 1:**
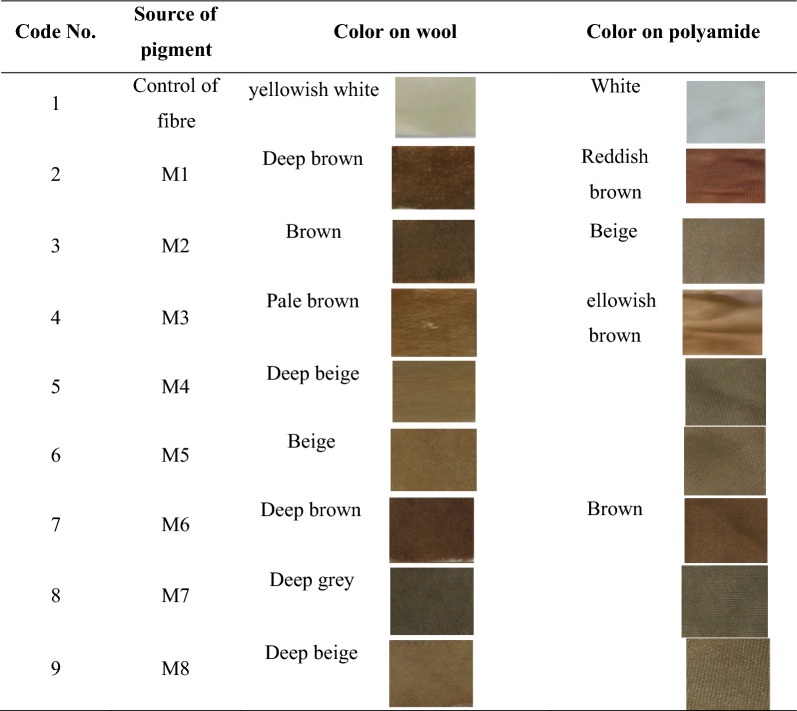
Dyeing of wool and polyamide using pigments produced by *Streptomyces torulosus*

Where M1 is yeast malt medium, M2 is modified yeast malt medium, M3 is tyrosine medium, M4 is modified tyrosine medium, M5 is modified tyrosine medium, M6 is glycerol asparagine medium, M7 is modified glycerol asparagine medium and M8 is modified glycerol asparagine medium

### Evaluation and characteristics of fabric color

The CIE L*, a*, and b* values for wool and polyamide 6 fabrics stained with the produced dyes from *Streptomyces torulosus* were detected. The letters L*, a*, and b* symbolize each of the 3 standards of the “CIELAB color space” utilized to identify color changes and measure color objectives. According to the L* value, which indicates perceived lightness or darkness from black to white, a degree of 0 denotes black and a degree of 100 denotes white [29]. From data tabulated in Table 2, the lightness degree varied between 48–63 and 46–66 for wool and polyamide, respectively. Based on that, the color of dyed textiles was fixed and distanced as indicated by Rosu et al. [40]. The values of a* and b* symbolize chromaticity without specific numeric limits. Positive a* corresponds with red, negative a* corresponds with green, positive b* corresponds with yellow, and negative b* corresponds with blue [28]. The results (Table 2) indicated that all dyed textile samples turned to red and yellow directions, and this was consistent with the results of Youssef et al., [41] who studied the dyeing of silk fabrics with natural pigments from two species of actinic bacteria.

**Table 2:** Color strength k/S and color coordinate L*, a* and b* of the dyed wool and polyamide fabrics

Samples	λmax		K/S		L*		a*		b*	
	W	P	W	P	W	P	W	P	W	P
M1	355	355	11.68	9.47	47.77	55.66	5.94	5.86	18.95	12.66
M2	355	355	6.33	5.45	57.19	65.81	5.79	3.31	19.08	1.73
M3	355	355	9.47	8.39	53.47	61.87	6.66	6.61	20.61	15.98
M4	355	370	4.24	7.40	62.87	58.05	3.83	2.71	17.80	11.30
M5	355	355	7.06	8.40	58.99	55.73	4.84	3.73	20.42	13.23
M6	355	355	10.36	14.59	49.25	46.40	7.55	6.85	18.17	16.88
M7	355	370	5.88	8.74	53.51	52.81	2.18	1.79	13.23	9.92
M8	355	355	6.99	7.82	59.44	58.10	4.93	3.44	19.99	10.57

Where; K/S, color strength; L*, Lightness; a*, Redness-Greenness of color; b*, Yellowness-Blueness of color; W, wool; P, polyamide

Measurements of perspiration and light fastness for the dyed samples during the washing process showed that only dyed wool fabrics demonstrated very good light fastness, while fastness to washing of the dyed samples rated as good to very good (Table S2 and S3). Therefore, the dyed samples can be considered to have “good to very good” fastness properties [41]. In the same context, Zhao et al. [28], in a similar study, demonstrated the stability of wool fabric printed using microbial dye extracted from *Streptomyces virginiae*, which showed a classification of fastness properties estimated as “good to very good”.

Chemically and physically, wool is heterogeneous, so dye absorption/uptake is assumed to occur in the intracellular areas of the cuticle (superficial) fiber layer (in the absence of damage to the cells) [42]. The wool fibers' intracellular region allows the dye to enter the fibers most easily. It then diffuses into the non-keratinous endocuticle zone of the surface layer, where it quickly reaches equilibrium with the dye in the outer solution. As the dye moves through the intracellular cement and enters the cells from their undersides, it first colors the endocuticle and then the exocuticle within the cuticle cells [43].

### Antimicrobial activity of natural pigment(s) produced by *Streptomyces torulosus*

The production of bioactive dyes with antimicrobial activity suitable for dyeing textiles for medical purposes was one of the main objectives of the present study. To investigate this goal, the produced pigments were used as antimicrobial agents against several pathogenic microbes. The potential antibacterial and antifungal compounds in the biosynthesized actinobacterial pigments were investigated. The targeted common pathogens were *S. cerevisiae*, *C. albicans*, and *A. niger* as fungi, *B. cereus,* and *S. aureus* as Gram-positive bacteria, and *E. coli* and *S. typhi* as Gram-negative bacteria. Unfortunately, all produced pigments didn^'^t have any inhibition effect on the tested pathogens. This may be due to the absence of antimicrobial compounds in the main structure of the produced pigments. However, this property can be improved using advanced new technologies such as nanotechnology [44]. In further studies, we try to enhance the bioactivity of these pigments using different tools such as the addition of silver nanoparticles to pigments for enhancing their antimicrobial activity.

### Production, characterization, and evaluation of the biological activity of AgNPs

In the previous section of this study, eight different dyes were obtained and evaluated for dyeing of 2 different textiles (wool and polyamide) and used as antimicrobial agents. For the first goal, the produced dyes have an extreme ability to dye the tested fabrics and result in different colors. Unfortunately, the second aim wasn't achieved in this section. For that, we try in the next section to enhance the properties of these dyes and try to obtain natural dyes that can dye textiles and inhibit pathogenic microbe growth.

**Fig. 3:**
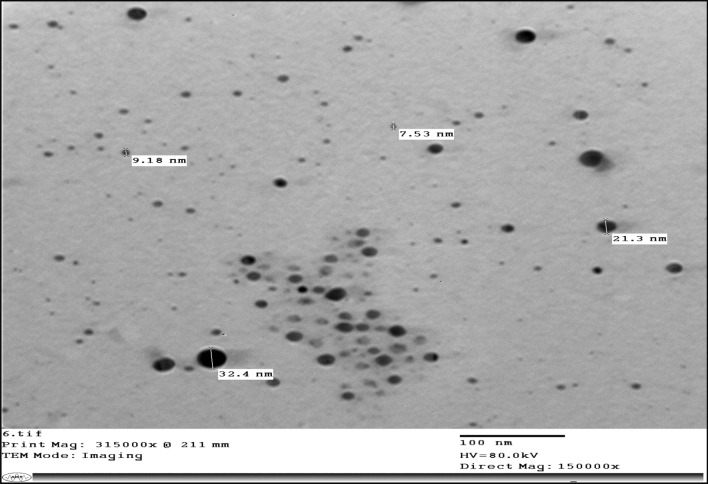
TEM characterization of the fungal-biosynthesized silver-NPs

*Fusarium oxysporum* as a fungus produces many metabolites substances as reducing agents [45] was used to produce silver nanoparticles. It was inoculated in potato dextrose broth medium and the filtrate was added to silver nitrate solution to form silver nanoparticles with a concentration of 485 µg/mL. After an overnight incubation period, the reaction mixture's color changed from pale yellow to brown, indicating the synthesis of silver nanoparticles. In many similar studies related to the biosynthesis of nanosilver, the formation of brown color in the reaction mixture was considered a clear indication of the transformation of silver ions into the metal nanoform [46]. The produced silver nanoparticles were characterized using a transmission electron microscope (TEM) and the results of the size and shape of produced nanoparticles were illustrated in Fig. 3. The size of these nanoparticles ranged between 10 and 30 nm, and the shape was spherical.

Particle size plays an essential role in determining the success of their adhesion to the fibers of the fabric on which they are to be immobilized. It is expected that larger (agglomerated) particles will be more easily removed from the fiber surface. While small particles can stabilize, they will penetrate deeper and adhere strongly to the tissue matrix [46]. Microbial synthesis of silver nanoparticles is considered an eco-friendly method for the production of nanomaterials. Because it produces less toxic and economical coast nanomaterials [47]. In this way, Ishida et al*.* [48]*,* produced 1.9–64.9 nm of silver nanoparticles using *Fusarium oxysporum* and used it as an antimicrobial agent against *C. glabrata, C. tropicalis, C. parapsilosis, C. krusei, C. albicans,* and *Cryptococcus sp.* pathogens.

The antimicrobial activity of silver nanoparticles (AgNPs) was evaluated against different microbial groups like Gram-positive and Gram-negative bacteria as well as yeast and fungi. The results for the antimicrobial activity of AgNPs were illustrated in Table 3 and Fig. (S1). The antimicrobial activity of biosynthesized silver nanoparticles at 485 µg/mL concentration against all tested pathogens ranged between 17 and 20 mm of inhibition zone diameter except the diameter of the well (6 mm). This indicates that the silver nanoparticles had biological activity as a result mentioned by Bamal et al*.* [49]*,* who found that antimicrobial activity of AgNPs (synthesized using *Escherichia coli*) against *Salmonella typhi*, *Bacillus subtilis*, *Klebsiella pneumoniae,* and *Vibrio cholera* was reached to 10–22 mm of inhibition diameter (at concentration of 250 ppm). Another author [50] found that AgNPs extracellular biosynthesized by *Fusarium acuminatum* at a concentration of 500 ppm showed effective antibacterial activity against *St. aureus*,* Sal. typhi*, *St. epidermidis,* and *E. coli* with 5–40 nm-diameter.

**Table 3:** Inhibition zone of antimicrobial activity of silver nanoparticles

Microbes		Inhibition zone (mm)
Gram-positive bacteria	*St. aureus*	20
	*B. cereus*	19.5
Gram-negative bacteria	*E. coli*	20
	*Sal. typhi*	20
Fungi	*C. albicans*	20
	*S. cereviciae*	17
	*A. niger*	17

### Enhancing the antimicrobial activity of the produced dyes by AgNPs combination

**Fig. 4:**
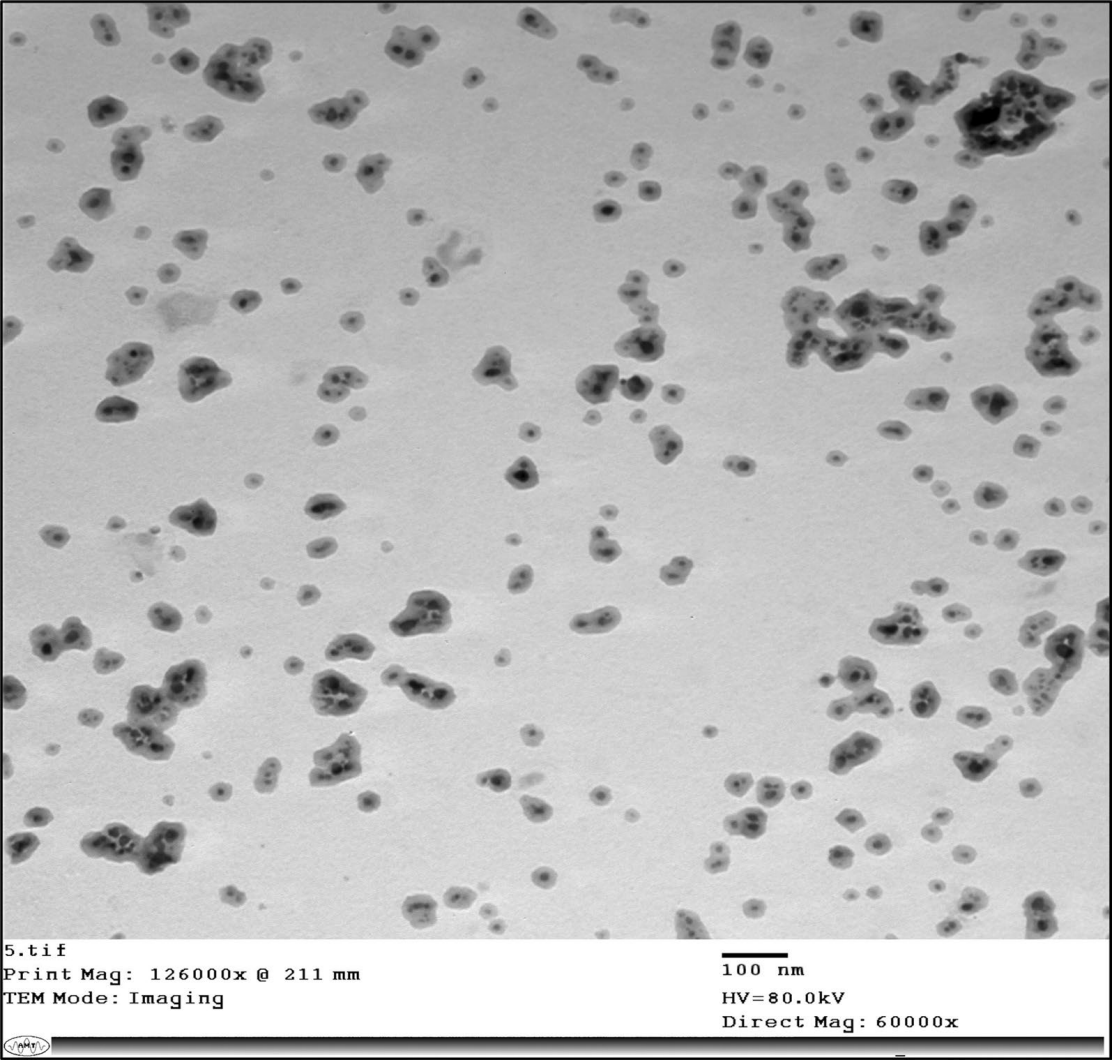
TEM image for AgNPs coated by natural dyes

In recent years, AgNPs used in various fields depending on their biological activity, especially in the textile approach; many researchers used them to enhance the fabric's bactericidal and fungicidal properties as the modified textiles in medical, sporting, environmental, or personal uses [51]. The application of AgNPs was in the form of adding nanoparticles to textile structure. However, in current research, AgNPs were added to natural dyes (as the first report) to modify dyes properties. This technology was the best dependent on entering AgNPs into the dye structure to produce a new composite as one material, this is clearly shown in Fig. 4. The image illustrated that AgNPs impeded into the dye structure, which means AgNPs coated by the produced natural dyes. It can be thought that the organic molecules coating AgNPs play a crucial role in the attachment of natural pigment with these particles making capsules.

The antimicrobial activity of natural dyes/AgNPs nanocomposite was determined against many pathogenic microbes. The nanocomposite produced from natural dyes and silver nanoparticles had AgNPs with a concentration of 243 µg/mL. The results represented in Table 4 and Fig. (S2) indicated that all tested nanocomposites had antimicrobial action against all examined pathogens and the high reaction reported by the nanocomposite resulted from dye symbolled M1 with AgNPs which had activity reached 18–20 mm of inhibition zone as illustrated in Fig. (S2). In this regard, the nanocomposites resulting from natural dyes symbolled M2, M5, M6, M7, and AgNPs showed a higher anti-gram-positive bacterial effect than Gram-negative bacteria. Also, the M1/AgNPs and M5/AgNPs nanocomposites represented high anti-candidal activity, while, M1/AgNPs and M4/AgNPs nanocomposites had anti-aspergillus activity (Table 4). The obtained results are considered the pioneer and the first report for recording the high antimicrobial activity of modified natural dyes. Ribeiro and his coworkers [52] stated that the addition of AgNPs to textiles were effective to inhibit the growth of *E. coli* and *St. aureus*.

**Table 4:** Inhibition zone for antibacterial and antifungal activity of the produced natural pigments supplemented by silver nanoparticles

Natural dyes/AgNPs nanocomposite	Inhibition zone (mm)						
	G + ve bacteria		G −ve bacteria		Fungi		
	*St. aureus*	*B. cereus*	*E. coli*	*Sal. typhi*	*C. albicans*	*S. cereviciae*	*A. niger*
H_2_O (control)	0	0	0	0	0	0	0
M_1_/AgNPs	19.5	19.0	20.1	18.5	20.0	18.0	19.5
M_2_/AgNPs	15.0	15.0	12.0	12.5	14.0	13.5	15.0
M_3_/AgNPs	15.5	15.0	15.0	16.0	15.0	13.0	15.5
M_4_/AgNPs	15.0	17.5	18.0	16.0	15.0	12.5	20.1
M_5_/AgNPs	17.0	17.0	14.5	14.0	18.5	12.5	17.0
M_6_/AgNPs	17.0	15.5	13.0	14.5	15.0	16.0	11.0
M_7_/AgNPs	17.0	16.0	14.5	14.0	15.0	16.0	13.5
M_8_/AgNPs	16.0	14.0	13.5	13.5	14.0	13.5	5.0

Where M1 is yeast malt medium, M2 is modified yeast malt medium, M3 is tyrosine medium, M4 is modified tyrosine medium, M5 is modified tyrosine medium, M6 is glycerol asparagine medium, M7 is modified glycerol asparagine medium and M8 is modified glycerol asparagine medium

### Application of natural dyes/AgNPs nanocomposites for textile dyeing and evaluation of their properties

The produced natural dyes/AgNPs nanocomposites as modified dyeing agents were applied to dye wool and polyamide fabrics. The properties of obtained dyed textiles were examined versus undyed controls to judge their biological and physicochemical properties. From textile properties, the biological activity toward pathogenic microorganisms is considered the main needed property in the special textile type [53]. The produced textile which dyed by dye/AgNPs nanocomposites as illustrated in Table 5 and Fig. (S3) was produced inhibition activity against the tested pathogens reached 7 mm as inhibition zone diameter over disc diameter. The promising inhibition activity of dyed fabrics toward pathogens may be due to the stabilization of the AgNPs into natural dye structure before using it for dyeing processes. The obtained results are in line with those reported by Broadhead et al. [54], who noted that the activity of textile after modification by metallic nanoparticles in inhibition of pathogens growth was developed. Eremenko et al. [55] found highly efficient bactericidal and antifungal properties of cotton fabrics containing silver and bimetallic nanoparticles as Ag/Cu composition (0.015–0.13 wt %). Their experiments confirmed positive results against a wide range of multidrug-resistant pathogens such as *E. coli, E. aerogenes, P. mirabilis, K. pneumoniae,* and *C. albicans*.

**Table 5:** Inhibition zone for antibacterial and antifungal activity of the produced natural pigments supplemented by silver nanoparticles

Textile discs of natural dyes/AgNPs nanocomposite	Inhibition zone* (mm)						
	G + ve bacteria		G −ve bacteria		Fungi		
	*St. aureus*	*B. cereus*	*E. coli*	*Sal. typhi*	*C. albicans*	*S. cereviciae*	*A. niger*
M1/AgNPs	2.0	2.5	3.5	3.5	2.0	2.5	0.0
M2/AgNPs	1.0	4.0	1.5	2.5	1.5	3.5	10
M3/AgNPs	3.5	2.0	3.5	7.0	5.5	6.0	0.0
M4/AgNPs	2.5	1.0	2.5	3.5	1.0	1.5	5.0
M5/AgNPs	1.0	4.5	2.0	2.5	1.0	1.0	7.5
M6/AgNPs	1.0	3.0	5.5	2.0	1.5	2.0	0.0
M7/AgNPs	2.5	3.0	4.5	4.0	7.5	1.5	0.0
M8/AgNPs	1.0	0.5	1.0	0.5	2.0	10.5	8.0

Where, * the diameter of effect determined without fabric diameter, M1 is yeast malt medium, M2 is modified yeast malt medium, M3 is tyrosine medium, M4 is modified tyrosine medium, M5 is modified tyrosine medium, M6 is glycerol asparagine medium, M7 is modified glycerol asparagine medium and M8 is modified glycerol asparagine medium

The physico-chemical properties of the dyed fabrics by dye/AgNPs nanocomposite were examined like color strength and color coordinate. In addition, the fastness properties i.e. washing, perspiration and light were also evaluated. The data illustrated in Table 6 showed that many colors appeared for the dyed textile by natural dye/AgNPs nanocomposite like brown, beige and grey. In practical field, especial health care sector, the produced colored textiles are suitable for hospital beds and/or clothes of hospitals stuff or workers. The obtained findings were agreed with results noted by Hoque et al., [56], who noted that the modified cotton fabrics by reactive organo-selenium displayed potent bactericidal action against *S. aureus* and moderate activity toward *E. coli*, and it acceptable for healthcare application in hospitals.

**Table 6:**
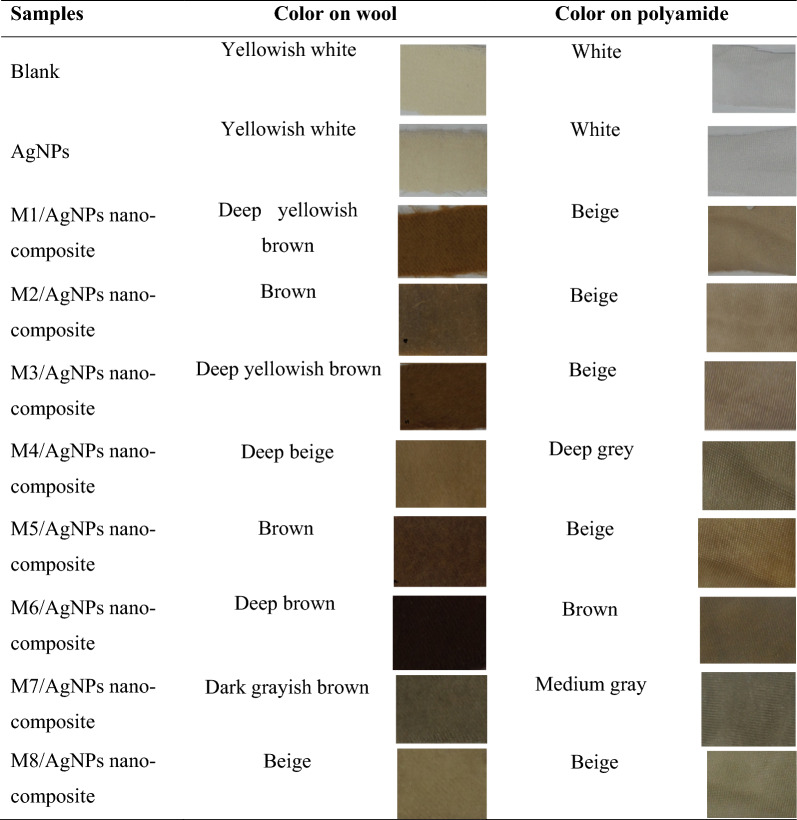
Color of fabrics after dyeing by natural dye/AgNPs nanocomposite

The CIE L*a*b* values for wool and polyamide fabrics dyed with colorants from *Strepotomyces torulosus* after modification by silver nanoparticles were presented in Table (S4 and S5). The results indicated that the K/S of dyed wool by nanocomposites of M1/AgNPs, M2/AgNPs, M3/AgNPs, M5/AgNPs and M6/AgNPs was increased and the color strength became higher than untreated one, while the L* was decreased in M1/AgNPs, M3/AgNPs, M5/AgNPs and M6/AgNPs. With another fabric, the K/S of dyed polyamide by nanocomposites of M1/AgNPs, M2/AgNPs, M3/AgNPs, M5/AgNPs and M6/AgNPs was decreased, while the K/S and L* of dyed polyamide by nanocomposites of M4/AgNPs, M7/AgNPs and M8/AgNPs didn’t change. The K/S refers to the color strength, so when the value of K/S increased indicates that the color is strong on the dyed samples [57]. On another side, the L* value, indicate perceived lightness or darkness, where 0 indicates black while 100 indicates white, so when the L* value decreased, this means that the color was dark but when the L* value increased this means that the color was light. Also, the value of a* indicates red (+ a) and green (−a), while b* signifies yellow (+ b) and blue (−b). The a* and b* values of dyed fabrics showed that all the fabrics dyed by the produced nanocomposites were found in the red–yellow zone. So, based on these results, we can conclude that the properties of dyed wool improved than the polyamide. In this trend, Abd El-Aziz et al. [58], found that the treated fabrics by silver nanoparticles had better K/S values than untreated ones. The higher color strength (K/S values) of nano-treated samples indicated that the presence of nanometal particles increases the dye affinity towards the textile material and the silver nanoparticles act as mordant in the fabric.

In the case of fastness properties of the dyed fabrics by natural dye/AgNPs nanocomposite, they were measured and the results indicated that dyed samples revealed “good to very good” fastness to washing (Table S6 and S7). This may be because the dyed fabrics have a great ability to keep the dyes entire their structure as in the washing process the excess of dyes in dyed samples was removed. Only dyed wool fabrics showed a very good rating of fastness to light. The dyed samples showed a “good to very good” rating for fastness properties, which is similar to the results obtained from fabrics dyed without AgNPs immobilization. In another study on dyed fabrics, Pizzicato et al. [3] found that there was no difference in the results of perspiration and light fastness tests in fabrics untreated or treated with Ag/TiO_2_ nanocomposite. While there was a slight decrease in washing stability and friction in dyed wool treated with the nanocomposite compared to untreated dyed wool. The authors mentioned that unwashed adhering dye particles on wool fabric may be a possible cause.

### Anticancer activity evaluation and cytotoxicity determination

The anticancer activity and cytotoxicity of the dyed fabrics were assessed using two human cell lines: the human normal fibroblast cell line (BJ1) and the human epidermoid carcinoma cell line (A431). Fortunately, the tested dyeing fabrics had anticancer properties against the A431 carcinoma cell line. Furthermore, the total count of cancer cells was decreased by 37% after treatment with the dyed fabrics. This finding supports the usage of the obtained modified textiles in the hospital branch. It is known, the hospitality clothes should have special characteristics like activity to inhibit pathogenic microbes and treat cancer cells. Also, the noted properties against cancer cell lines were advantaged by cytotoxicity of the tested samples, because they hadn’t any cytotoxicity against BJ1 cell line. The results obtained by normal cell line cleared show the colored textile did not cause decrease the total viable cell count after incubation period.

## Conclusion

The natural Streptomyces-pigments were produced by *Streptomyces torulosus* isolate OSh10 (KX753680.1) after optimization of culture conditions. The produced pigments enhanced their properties by myco-synthesized AgNPs (spherical with around 10 nm size). Also, the obtained modified textiles can be used in hospitals to protect patients from pathogenic microbes as well as it is suitable with cancer patients. So, the current study fills the gap in human health areas using environmentally safe agents.

### Supplementary Information

Supplementary material 1.

## Data Availability

Data will be available on request.

## References

[1] Health and Safety Executive (HSE). Dyes and Chemicals in Textile Finishing: An introduction. Dyeing and Finishing Information Sheet No 1-HSE Information Sheet. 2016. https://www.hse.gov.uk/textiles/dyes-dyeing.htm. Accessed 4 Nov 2023.

[2] Hassaan MA, Nemr AE (2017). Health and environmental impacts of dyes: mini review. Am J Environ Sci Eng.

[3] Pizzicato B, Pacifico S, Cayuela D, Mijas G, Riba-Moliner M (2023). Advancements in sustainable natural dyes for textile applications: a review. Molecules.

[4] Çelik Yilmaz N, Yilmaz A, Yilmaz F (2023). Coloring of woolen fabrics with natural resources and investigating the color perceptions of children on these fabrics. J Nat Fibers.

[5] Mouro C, Gomes AP, Costa RV, Moghtader F, Gouveia IC (2023). The sustainable bioactive dyeing of textiles: a novel strategy using bacterial pigments, natural antibacterial ingredients, and deep eutectic solvents. Gels.

[6] Affat SS (2021). Classifications, advantages, disadvantages, toxicity effects of natural and synthetic dyes: a review. Univ Thi-Qar J Sci.

[7] Bahtiyari Mİ, Yilmaz F (2018). Evaluation of different natural dye sources in terms of metamerism. AATCC J Res.

[8] Che J, Yang X (2022). A recent (2009–2021) perspective on sustainable color and textile coloration using natural plant resources. Heliyon.

[9] Lara L, Cabral I, Cunha J (2022). Ecological approaches to textile dyeing: a review. Sustainability.

[10] Yılmaz F, Bahtiyari Mİ (2022). An approach for linen fabrics coloring and antibacterial activity by cumin in combination with nano copper and iron. J Nat Fibers.

[11] Darwesh OM, Barakat KM, Mattar MZ, Sabae SZ, Hassan SH (2019). Production of antimicrobial blue green pigment Pyocyanin by marine *Pseudomonas aeruginosa*. Biointerf Res Appl Chem.

[12] Azman AS, Mawang CI, Abu BS (2018). Bacterial pigments: the bioactivities and as an alternative for therapeutic applications. Nat Prod Comm.

[13] Agarwal H, Bajpai S, Mishra A, Kohli I, Varma A, Fouillaud M, Dufossé L, Joshi NC (2023). Bacterial pigments and their multifaceted roles in contemporary biotechnology and pharmacological applications. Microorganisms.

[14] Siddiqua UH, Zaib-un-Nisa RA (2024). Effect of silver nanoparticles finishing on dyeing properties of newly synthesized reactive dye applied on cellulosic fabric. Fibers Polym.

[15] Zhang F, Wu X, Chen Y (2009). Application of silver nanoparticles to cotton fabric as an antibacterial textile finish. Fibers Polym.

[16] Van Der Kraan M (2007). Equilibrium study on the disperse dyeing of polyester textile in supercritical carbon dioxide. Text Res J.

[17] Rahman NA, Tajuddin R, Tumin S (2013). Optimization of natural dyeing using ultrasonic method and biomordant. Int J Chem Eng Appl.

[18] Orylska-Ratynska M, Placek W, Owczarczyk-Saczonek A (2022). Tetracyclines-an important therapeutic tool for dermatologists. Int J Environ Res Public Health.

[19] Bidell MR, Pai MP, Lodise TP (2020). Use of oral tetracyclines in the treatment of adult patients with community-acquired bacterial pneumonia: a literature review on the often-overlooked antibiotic class. Antibiotics.

[20] do Barreto JV, Casanova LM, Junior AN, Reis-Mansur MCPP, Vermelho AB (2023). Microbial pigments: major groups and industrial applications. Microorganisms.

[21] Kheiralla ZH, Hewedy MA, Mohammed HR, Darwesh OM (2016). Isolation of pigment producing actinomycetes from rhizosphere soil and application it in textiles dyeing. J Pharm Biol Chem Sci.

[22] Mourad R, Helaly F, Darwesh OM, Sawy SE (2019). Antimicrobial and physicomechanical natures of silver nanoparticles incorporated into silicone- hydrogel films. Cont Lens Anterior Eye.

[23] El-Shanshoury AR, Darwesh OM, Sabae SZ, Awadallah OA, Hassan SH (2020). Bio-manufacturing of selenium nanoparticles by *Bacillus subtilis* isolated from Qarun Lake and evaluation their activity for water remediation. Biointerf Res Appl Chem.

[24] Mourad RM, Darwesh OM, Abdel-Hakim A (2020). Enhancing physico-mechanical and antibacterial properties of natural rubber using synthesized Ag-SiO_2_ nanoparticles. Int J Biol Macromol.

[25] Abdelhameed RM, Darwesh OM, El-Shahat M (2020). Synthesis of arylidene hydrazinylpyrido[2,3-d]pyrimidin-4-ones as potent anti-microbial agents. Heliyon.

[26] Mosaad RM, Alhalafi MH, Emam E-AM, Ibrahim MA, Ibrahim H (2022). Enhancement of antimicrobial and dyeing properties of cellulosic fabrics via chitosan nanoparticles. Polymers.

[27] Baaka N, Khiari R, Haji A (2023). Ecofriendly dyeing of textile materials with natural colorants from date palm fiber fibrillium. Sustainability.

[28] Zhao Z, Yan C, Xu F, Liu J (2023). Study on dyeing properties and color characteristics of wool fabrics dyed with *Geranium caespitosum* L. extract- a new natural yellow dye. Coatings.

[29] Kumpikaitė E, Tautkutė-Stankuvienė I, Milašienė D, Petraitienė S (2022). Analysis of color fastness and shrinkage of dyed and printed linen/silk fabrics. Coatings.

[30] Mabrouk AM, El-khrisy EAM, Youssef YA, Asem AM (2011). Production of textile reddish brown dyes by fungi. Malays J Microbiol.

[31] Repetto G, Del Peso A, Zurita JL (2008). Neutral red uptake assay for the estimation of cell viability/cytotoxicity. Nat Proto.

[32] Darwesh OM, Eweys AS, Zhao YS, Matter IA (2023). Application of environmental-safe fermentation with *Saccharomyces cerevisiae* for increasing the cinnamon biological activities. Bioresour Bioproc.

[33] Abd El-Fattah NM. Bioleaching and biosorption of some rare earth elements and actinides from soil sample in Sinai. MSC Thesis, Ain shams university, Cairo, 2012; 180 pp.

[34] Schmidt FR (2005). Optimization and scale up of industrial fermentation processes. Appl Microbiol Biotechnol.

[35] Hewedy MA, Ashour SM (2009). Production of a melanin like pigment by *Kluyveromyces marxianus* and *Streptomyces chibaensis*. Aust J Basic Appl Sci.

[36] Pandey R, Chander R, Sainis KB (2007). Prodigiosins: a novel family of immunosuppressants with anticancer activity. Indian J Biochem Biophys.

[37] Abussaud MJ, Alanagreh L, Abu-Elteen K (2013). Isolation, characterization and antimicrobial activity of *Streptomyces* strains from hot spring areas in the northern part of Jordan. Afr J Biotechnol.

[38] Abo El-Ola SM (2008). Recent developments in finishing of synthetic fibers for medical applications. Des Monomers Polym.

[39] Abo El-Ola SM (2007). New approach for imparting antimicrobial properties for polyamide and wool containing fabrics. Polym Plast Technol Eng.

[40] Rosu L, Gavat C-C, Rosu D, Varganici C-D, Mustata F (2021). Photochemical stability of a cotton fabric surface dyed with a reactive triphenodioxazine dye. Polymers.

[41] Yusoff WFW, Mohamad SAS, Ahmad WYW, Ahmad MR, Yahya MF (2014). Fastness properties and color analysis of natural colorants from actinomycetes isolates on silk fabric. Proceedings of the international colloquium in textile engineering.

[42] Joko K, Koga J. Proc. 9th Internat. Wool *Text. Res*. Conference, 1990; 19–26.

[43] Musnickas J, Rupainyte V, Treigiene R, Rageliene L (2005). Dye migration influences on color characteristics of wool fabric dyed with acid dye. Fibres Text East Eur.

[44] Darwesh OM, Al-Balakocy NG, Ghanem A, Matter IA (2023). Application of microalgal-ZnO-NPs for reusing polyester/cotton blended fabric wastes after modification by cellulases enzymes. Waste Dispos Sustain Energy.

[45] Darwesh OM, Li H, Matter IA (2023). Nano-bioremediation of textile industry wastewater using immobilized CuO-NPs myco-synthesized by a novel Cu-resistant *Fusarium oxysporum* OSF18. Environ Sci Pollut Res.

[46] Namasivayam SKR (2011). Silver nanoparticle synthesis from lecanicillium lecanii and evolutionary treatment on cotton fabrics by measuring their improved antibacterial activity with antibiotics against *staphylococcus aureus* (ATCC 29213) and *E. coli* (ATCC 25922) strains. Int J Pharm Sci.

[47] Abdel-Hadi A, Iqbal D, Alharbi R, Jahan S, Darwish O, Alshehri B, Banawas S, Palanisamy M (2023). Myco-synthesis of silver nanoparticles and their bioactive role against pathogenic microbes. Biology.

[48] Ishida K, Cipriano TF, Rocha GM, Weissmüller G, Gomes F, Miranda K, Rozental S (2014). Silver nanoparticle production by the fungus *Fusarium oxysporum*: nanoparticle characterisation and analysis of antifungal activity against pathogenic yeasts. Mem Inst Oswaldo Cruz.

[49] Bamal D, Singh A, Chaudhary G, Kumar M, Singh M, Rani N, Mundlia P, Sehrawat AR (2021). Silver nanoparticles biosynthesis, characterization, antimicrobial activities, applications, cytotoxicity and safety issues: an updated review. Nanomaterials.

[50] Ingle A, Gade A, Pierrat S, Sonnichsen C, Rai M (2008). Mycosynthesis of silver nanoparticles using the fungus *Fusarium acuminatum* and its activity against some human pathogenic bacteria. Curr Nanosci.

[51] Srinivas K (2016). The role of nanotechnology in modern textiles. J Chem Pharm Res.

[52] Ribeiro AI, Shvalya V, Cvelbar U, Silva R, Marques-Oliveira R, Remião F, Felgueiras HP, Padrão J, Zille A (2022). Stabilization of silver nanoparticles on polyester fabric using organo-matrices for controlled antimicrobial performance. Polymers.

[53] Tanasa F, Teaca C-A, Nechifor M, Ignat M, Duceac IA, Ignat L (2023). Highly specialized textiles with antimicrobial functionality-advances and challenges. Textiles.

[54] Broadhead R, Craeye L, Callewaert C (2021). The future of functional clothing for an improved skin and textile microbiome relationship. Microorganisms.

[55] Eremenko AM, Petrik IS, Smirnova NP, Rudenko AV, Marikvas YS (2016). Antibacterial and antimycotic activity of cotton fabrics, impregnated with silver and binary silver/copper nanoparticles. Nanoscale Res Lett.

[56] Hoque E, Tran P, Jacobo U, Bergfeld N, Acharya S, Shamshina JL, Reid TW, Abidi N (2023). Antimicrobial coatings for medical textiles via reactive organo-selenium compounds. Molecules.

[57] Tang YLA, Jin S, Lee CH, Law HS, Yu J, Wang Y, Kan C-W (2023). Reverse micellar dyeing of cotton fabric with reactive dye using biodegradable non-ionic surfactant as nanoscale carrier: an optimization study by one-factor-at-one-time approach. Polymers.

[58] Abd El-Aziz E, Zayed M, Mohamed AL, Hassabo AG (2023). Enhancement of the functional performance of cotton and polyester fabrics upon treatment with polymeric materials having different functional groups in the presence of different metal nanoparticles. Polymers.

