# Supplementary material for: Natural dyes developed by microbial-nanosilver to produce antimicrobial and anticancer textiles
Source: Microb Cell Fact. 2024 Jul 2;23:189. doi: 10.1186/s12934-024-02457-3 (PMC11218209; doi:10.1186/s12934-024-02457-3)
Supplement: Supplementary file 1 — Supplementary material 1. [file 12934_2024_2457_MOESM1_ESM.docx]

**Supplementary data**


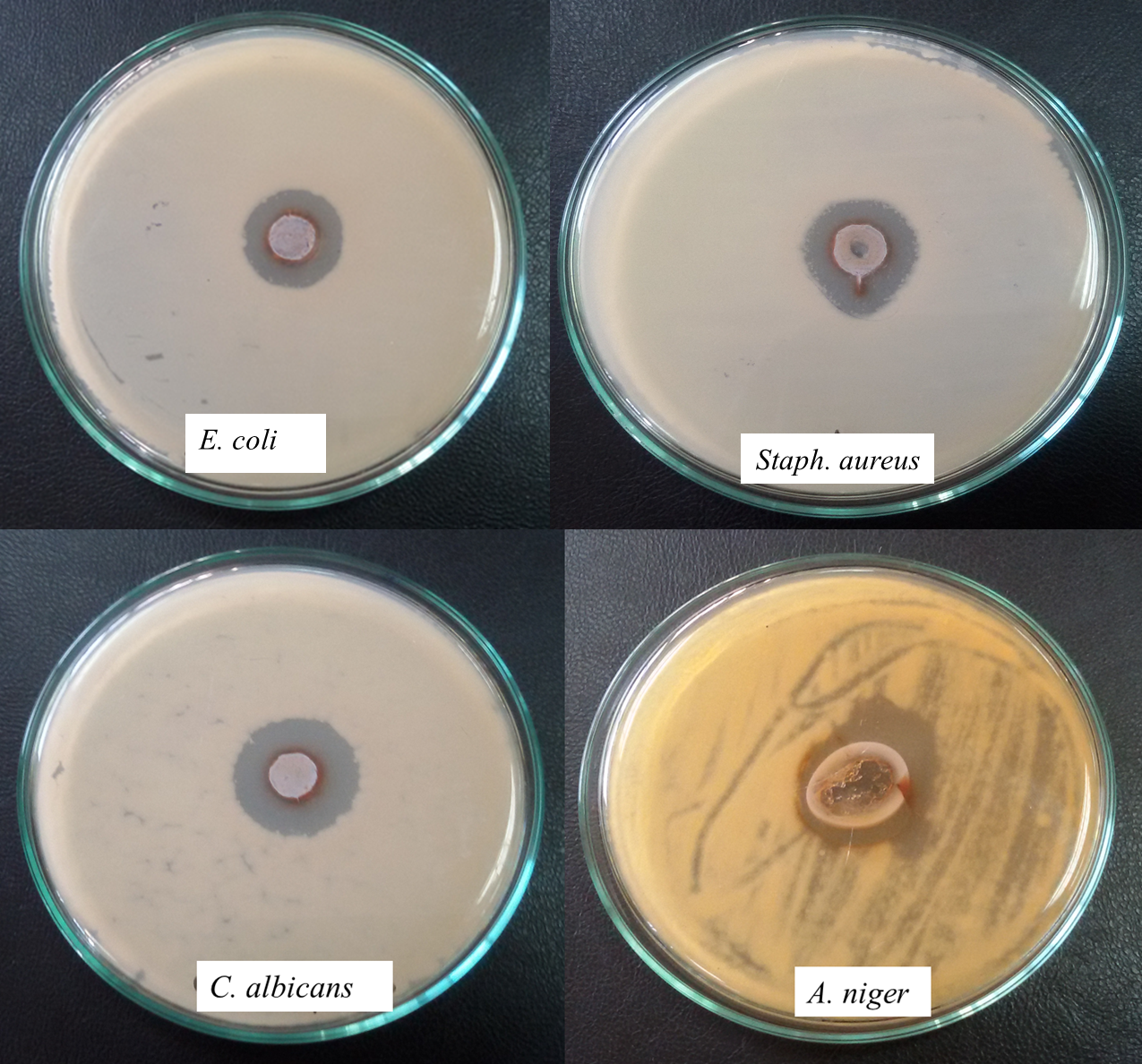


**Fig. S1.** Inhibition zone of antibacterial activity of AgNPs against *Staph. Aureus, E. coli, A. niger* and *C. albicans*

**
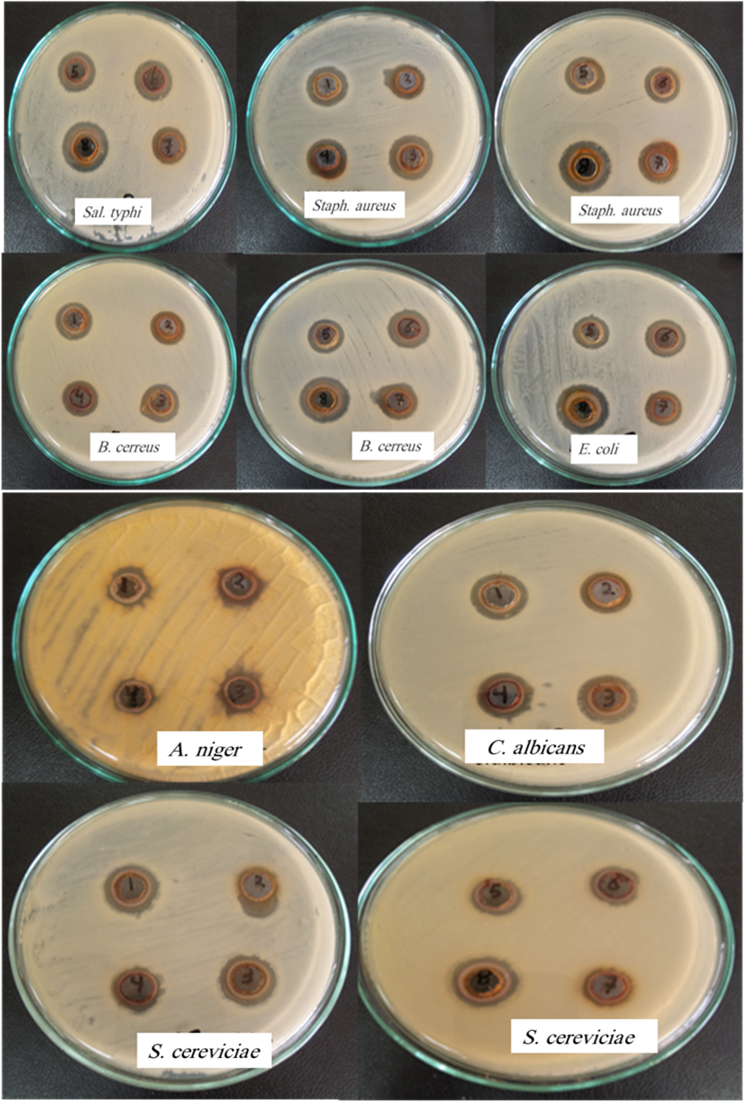
**

**Fig. S2.** Inhibition zone for antibacterial and antifungal activity of dyes enhanced by AgNPs where 1 as M_7_, 2 as M_8_, 3 as M_6_, 4 as M_2_, 5 as M_3_, 6 as M_4_, 7 as M_5_, 8 as AgNPs

**Table S1.** Optimization of carbon and nitrogen sources for natural dye production by *S. torulosus*

|  | **Media** | | |
| --- | --- | --- | --- |
|  | **Yeast malt (ISP2)** | **Glycerol Asparagine (ISP5)** | **Tyrosine (ISP7)** |
| **λ_max_ (nm)** | 525 | 380 | 390 |
| **Main carbon source** | Dextrose | Glycerol | Glycerol |
| **Tested carbon source** | Sucrose, Lactose, Glycerol and Starch | Dextrose, Sucrose, Lactose and Starch | Dextrose, Sucrose, Lactose and Starch |
| **Main nitrogen source** | Malt extract | L-asparagine | L-asparagine and L-tyrosine |
| **Tested nitrogen source** | L-asparagine, Peptone, NaNO_3_, (NH_4_)_2_SO_4_ | L-tyrosine, Peptone, NaNO_3_, (NH_4_)_2_SO_4_ | Yeast extract, Peptone, NaNO_3_, (NH_4_)_2_SO_4_ |

**Table S2.** Washing, perspiration and light fastness properties of the dyed wool

| **Sample** | **Fastness to washing** | | | **Fastness to perspiration** | | | | | | **Light** |
| --- | --- | --- | --- | --- | --- | --- | --- | --- | --- | --- |
|  |  |  |  | **Acidic perspiration** | | | **Alkaline perspiration** | | |  |
|  | **Alt.** | **SC** | **SW** | **Alt.** | **SC** | **SW** | **Alt.** | **SC** | **SW** |  |
| **M1** | 4-5 | 4-5 | 4 | 4-5 | 5 | 5 | 4-5 | 4-5 | 4-5 | 5 |
| **M2** | 5 | 5 | 5 | 5 | 5 | 5 | 5 | 5 | 5 | 3 |
| **M3** | 4-5 | 4-5 | 4 | 4-5 | 4-5 | 4-5 | 5 | 5 | 5 | 4-5 |
| **M4** | 4-5 | 5 | 4-5 | 4-5 | 5 | 4-5 | 4-5 | 5 | 4-5 | 3-4 |
| **M5** | 4-5 | 4-5 | 4-5 | 4-5 | 5 | 4-5 | 4-5 | 5 | 5 | 3-4 |
| **M6** | 5 | 4-5 | 4-5 | 5 | 5 | 5 | 4-5 | 5 | 4-5 | 4-5 |
| **M7** | 5 | 5 | 4-5 | 5 | 5 | 4-5 | 5 | 4-5 | 4-5 | 4-5 |
| **M8** | 4 | 4-5 | 4 | 4-5 | 4-5 | 4-5 | 5 | 5 | 5 | 3 |

**Where; Alt, Alteration of the dye yield of stained wool in comparison with non-treated one; SC, staining on non-dyed cotton; SW, staining on non-dyed wool.**

**Table S3.** Fastness properties (washing, perspiration and light) of the dyed polyamide

| **Sample** | **Fastness to washing** | | | **Fastness to perspiration** | | | | | | **Light** |
| --- | --- | --- | --- | --- | --- | --- | --- | --- | --- | --- |
|  |  |  |  | **Acidic perspiration** | | | **Alkaline perspiration** | | |  |
|  | **Alt.** | **SC** | **SW** | **Alt.** | **SC** | **SW** | **Alt.** | **SC** | **SW** |  |
| **M1** | 4-5 | 4-5 | 4 | 4-5 | 4-5 | 4-5 | 4-5 | 4-5 | 4-5 | 2-3 |
| **M2** | 4-5 | 4-5 | 4-5 | 4-5 | 4-5 | 4-5 | 4-5 | 5 | 4-5 | 3-4 |
| **M3** | 4-5 | 4-5 | 4-5 | 4-5 | 4-5 | 4-5 | 4-5 | 4-5 | 4-5 | 2-3 |
| **M4** | 4-5 | 4-5 | 4-5 | 4-5 | 4-5 | 4-5 | 5 | 5 | 5 | 3-4 |
| **M5** | 4-5 | 5 | 4-5 | 4-5 | 5 | 5 | 4-5 | 5 | 4-5 | 3-4 |
| **M6** | 4-5 | 4-5 | 4-5 | 5 | 5 | 5 | 5 | 5 | 5 | 3 |
| **M7** | 4-5 | 4-5 | 4 | 5 | 5 | 5 | 5 | 5 | 5 | 3 |
| **M8** | 5 | 4-5 | 4-5 | 5 | 5 | 5 | 4-5 | 5 | 4-5 | 3 |

**Where; Alt: Alteration of the dye yield of stained polyamide in comparison with non-treated one; SC, staining on non-dyed cotton; SW, staining on non-dyed wool.**

**Table S4.** Color strength (k/S) and color coordinate (L*a*b*) of the dyed wool by dyes/AgNPs nanocomposite

| **Sample** | **λmax.** | **K/S** | **L*** | **a*** | **b*** |
| --- | --- | --- | --- | --- | --- |
| **M_1_/AgNPs** | 355 | 15.07 | 47.15 | 11.51 | 29.63 |
| **M_2_/AgNPs** | 365 | 9.24 | 53.75 | 4.98 | 21.27 |
| **M_3_/AgNPs** | 365 | 16.50 | 40.28 | 10.31 | 24.62 |
| **M_4_/AgNPs** | 355 | 4.33 | 62.90 | 3.87 | 17.83 |
| **M_5_/AgNPs** | 370 | 14.11 | 39.06 | 12.66 | 24.21 |
| **M_6_/AgNPs** | 365 | 21.88 | 23.17 | 11.50 | 11.59 |
| **M_7_/AgNPs** | 355 | 5.86 | 53.54 | 2.20 | 13.25 |
| **M_8_/AgNPs** | 355 | 6.75 | 59.48 | 4.97 | 20.5 |

**Where; K/S, color strength; L*, Lightness; a*, Redness-Greenness of color; b*, Yellowness-Blueness of color**

**Table S5.** Color strength (k/S) and color coordinate (L*a*b*) of the dyed polyamide by dyes/AgNPs nanocomposite

| **Sample** | **λmax.** | **K/S** | **L*** | **a*** | **b*** |
| --- | --- | --- | --- | --- | --- |
| **M_1_/AgNPs** | 355 | 4.05 | 67.60 | 3.53 | 7.81 |
| **M_2_/AgNPs** | 355 | 3.52 | 65.72 | 3.15 | 6.34 |
| **M_3_/AgNPs** | 370 | 3.62 | 66.12 | 3.65 | 8.90 |
| **M_4_/AgNPs** | 370 | 7.45 | 58.10 | 2.75 | 11.36 |
| **M_5_/AgNPs** | 355 | 5.39 | 61.82 | 4.43 | 10.49 |
| **M_6_/AgNPs** | 370 | 8.74 | 53.51 | 2.08 | 22.61 |
| **M_7_/AgNPs** | 370 | 8.79 | 52.85 | 1.82 | 9.96 |
| **M_8_/AgNPs** | 355 | 7.86 | 58.15 | 3.47 | 10.60 |

**Where; K/S, color strength; L*, Lightness; a*, Redness-Greenness of color; b*, Yellowness-Blueness of color**

**Table S6.** Washing, perspiration and light fastness properties of the dyed wool by dyes/AgNPs nanocomposite

| **Samples** | **Fastness to washing** | | | **Fastness to perspiration** | | | | | | **Light** |
| --- | --- | --- | --- | --- | --- | --- | --- | --- | --- | --- |
|  |  |  |  | **acidic perspiration** | | | **alkaline perspiration** | | |  |
|  | **Alt.** | **SC** | **SW** | **Alt.** | **SC** | **SW** | **Alt.** | **SC** | **SW** |  |
| **M_1_/AgNPs** | 5 | 4-5 | 4-5 | 4-5 | 5 | 5 | 4-5 | 4-5 | 4-5 | 3-4 |
| **M_2_/AgNPs** | 4-5 | 4-5 | 5 | 4-5 | 4 | 4-5 | 4-5 | 4 | 4-5 | 3 |
| **M_3_/AgNPs** | 5 | 4-5 | 4-5 | 4-5 | 4-5 | 4-5 | 5 | 4 | 4-5 | 4-5 |
| **M_4_/AgNPs** | 4-5 | 5 | 4-5 | 4-5 | 5 | 4-5 | 4-5 | 5 | 4-5 | 3-4 |
| **M_5_/AgNPs** | 4-5 | 4-5 | 4-5 | 4-5 | 4 | 4-5 | 4-5 | 4 | 4-5 | 4 |
| **M_6_/AgNPs** | 5 | 4-5 | 4-5 | 5 | 4 | 4 | 5 | 4 | 4 | 4-5 |
| **M_7_/AgNPs** | 5 | 5 | 4-5 | 5 | 5 | 4-5 | 5 | 4-5 | 4-5 | 4-5 |
| **M_8_/AgNPs** | 4 | 4-5 | 4 | 4-5 | 4-5 | 4-5 | 5 | 5 | 5 | 3 |

**Where; Alt, Alteration of the dye yield of dyed wool and poly amide in comparison with non-treated one; SC, Staining on non-dyed cotton; SW, Staining on non-dyed wool**

**Table S7.** Washing, perspiration and light fastness properties of the dyed polyamide by dyes/AgNPs nanocomposite

| **Samples** | **Fastness to washing** | | | **Fastness to perspiration** | | | | | | **Light** |
| --- | --- | --- | --- | --- | --- | --- | --- | --- | --- | --- |
|  |  |  |  | **acidic perspiration** | | | **Alkaline perspiration** | | |  |
|  | **Alt.** | **SC** | **SW** | **Alt.** | **SC** | **SW** | **Alt.** | **SC** | **SW** |  |
| **M_1_/AgNPs** | 4-5 | 5 | 4-5 | 4-5 | 5 | 5 | 4 | 4-5 | 4-5 | 3 |
| **M_2_/AgNPs** | 4-5 | 5 | 5 | 4-5 | 4-5 | 4-5 | 4-5 | 4 | 4-5 | 3 |
| **M_3_/AgNPs** | 4-5 | 4-5 | 4-5 | 4 | 4-5 | 4-5 | 4 | 4-5 | 4-5 | 3 |
| **M_4_/AgNPs** | 4-5 | 4-5 | 4-5 | 4-5 | 4-5 | 4-5 | 5 | 5 | 5 | 3-4 |
| **M_5_/AgNPs** | 4-5 | 5 | 4-5 | 4 | 4-5 | 5 | 4-5 | 4-5 | 4-5 | 3-4 |
| **M_6_/AgNPs** | 5 | 5 | 5 | 4-5 | 4-5 | 4-5 | 4-5 | 4-5 | 4-5 | 4-5 |
| **M_7_/AgNPs** | 4-5 | 4-5 | 4 | 5 | 5 | 5 | 5 | 5 | 5 | 3 |
| **M_8_/AgNPs** | 5 | 4-5 | 4-5 | 5 | 5 | 5 | 4-5 | 5 | 4-5 | 3 |

**Where; Alt, Alteration of the dye yield of dyed wool and poly amide in comparison with non-treated one; SC, Staining on non-dyed cotton; SW, Staining on non-dyed wool**
